# The Physical Activity Assessment of Adults With Type 2 Diabetes Using Accelerometer-Based Cut Points: Scoping Review

**DOI:** 10.2196/34433

**Published:** 2022-09-06

**Authors:** Ioana A Moldovan, Alexa Bragg, Anna S Nidhiry, Barbara A De La Cruz, Suzanne E Mitchell

**Affiliations:** 1 Department of Family Medicine Boston Medical Center Boston, MA United States; 2 Department of Family Medicine Boston University School of Medicine Boston, MA United States; 3 Department of Family Medicine and Community Health University of Massachusetts Chan Medical School Worcester, MA United States

**Keywords:** accelerometer, cut points, type 2 diabetes, physical activity

## Abstract

**Background:**

Incorporating physical activity into lifestyle routines is recommended for individuals with type 2 diabetes. Accelerometers offer a promising method for objectively measuring physical activity and for assessing interventions. However, the existing literature for accelerometer-measured physical activity among middle-aged and older adults with type 2 diabetes is lacking.

**Objective:**

This study aims to identify research studies in which accelerometer-based cut points were used to classify the physical activity intensity of middle-aged to older adults with type 2 diabetes as sedentary, light, moderate, vigorous, and very vigorous, and to determine if validated accelerometer cut points specifically for this population exist.

**Methods:**

We followed the Joanna Briggs Institute methodology for scoping reviews. Between June 23 and July 12, 2020, two reviewers independently screened records from four databases (PubMed, Web of Science, Embase, Engineering Village) and the ActiGraph Corp web site for eligible studies that included patients with type 2 diabetes with a sample mean age ≥50 years, used research-grade accelerometers, applied cut points to categorize objectively measured physical activity, and were available in English. We excluded studies reporting exclusively steps or step counts measured by accelerometers or pedometers and conference abstracts or other sources that did not have a full text available. Data extraction was completed using Microsoft Excel. Data for the following variables were tabulated based on frequency distributions: study design, accelerometer type, device placement, epoch length, total wear time, and cut points used. Study aims and participant demographic data were summarized.

**Results:**

A total of 748 records were screened at the abstract level, and 88 full-text articles were assessed for eligibility. Ultimately, 46 articles were retained and analyzed. Participants’ mean ages ranged from 50 to 79.9 years. The ActiGraph accelerometer and the Freedson et al and Troiano et al counts-per-minute cut points were the most frequently used across the literature. Freedson et al and Troiano et al counts-per-minute cut points for light, moderate, and vigorous activity correspond to <1952, 1952-5724, and ≥5725, and 100-2019, 2020-5998, and ≥5999, respectively. The Lopes et al cut points were developed by calibrating the ActiGraph in middle-aged and older adults with overweight/obesity and type 2 diabetes. These counts-per-minute thresholds are ≥200 (light), ≥1240 (moderate), and ≥2400 (vigorous), and were applied in 1 interventional study.

**Conclusions:**

An assortment of accelerometer cut points have been used by researchers to categorize physical activity intensity for middle-aged and older adults with diabetes. Only one set of cut points was validated and calibrated in our population of interest. Additional research is warranted to address the need for diabetes-specific cut points to inform public health recommendations. This includes confirmation that the Lopes et al cut points reflect clinically meaningful changes in physical activity for adults with diabetes who have comorbidities other than overweight/obesity and the development of relative intensity cut points that may be more suitable for those with suboptimal physical functioning.

## Introduction

### Background

Approximately 462 million individuals are affected by type 2 diabetes (T2D) globally [1], and the vast majority of the estimated 37 million Americans with diabetes have T2D [2]. Physical inactivity is a known risk factor for diabetes complications, yet about 38% of adults with diabetes achieve less than 10 minutes per week of moderate or vigorous activity [3]. These individuals generally engage in lower levels of physical activity duration and intensity [4], and have lower physical functional capacity compared to adults without diabetes [5].

### Rationale

While many interventions for individuals with diabetes promote reducing sedentary time by increasing physical activity, accurate assessment of physical activity remains a challenge. Self-report questionnaires have traditionally quantified physical activity in numerous research studies but are subject to recall bias and lack of standardization [6]. In the last decade, accelerometers have shown promise for their ability to objectively measure the body’s acceleration in at least one of three orthogonal planes (anteroposterior, mediolateral, and vertical) and convert it into activity counts, also reported as counts per minute. These counts, which are proportional to the amount of physical activity performed by an accelerometer wearer, can be further used to categorize exercise into intensity levels (light, moderate, vigorous) [7]. Intensity thresholds, or cut points, have commonly been determined from regression equations relating accelerometer-recorded counts per minute and simultaneous measurement of energy expenditure (as metabolic equivalents of task [METs]) during laboratory and free-living activities [8].

For people with T2D, exercise can help lower blood glucose levels, lipid levels, and blood pressure, thus improving diabetes outcomes [9]. Several population-specific thresholds have been established to define physical activity intensity using accelerometers such as the ActiGraph monitors, which comprise ≥50% of activity monitors globally [10]. Existing reviews related to this topic are limited to only consumer-wearable activity trackers [11], randomized controlled trials (RCTs) [12,13], or walking measured as steps per day [14,15]. Determining the most appropriate protocols for capturing accelerometer data in this population to accurately inform disease-specific public health recommendations is critical. This scoping review is comprehensive and aims to describe the use of accelerometer-based cut points for assessing physical activity intensity of adults with T2D.

### Review Questions

We address two questions: (1) which cut points, if any, have been used to objectively categorize the physical activity intensity of adults with T2D (mean age ≥50 years) and (2) do accelerometer thresholds specifically validated for this population exist in the literature?

## Methods

This scoping review was conducted following the Joanna Briggs Institute Manual for Evidence Synthesis [16] using the following protocol: a limited search of relevant databases with an analysis of title and abstract keywords, and of article index terms; a comprehensive search using identified keywords and index terms across all databases; and a search of the reference lists of all full-text articles included in the review.

### Inclusion Criteria for Sources of Evidence

The criteria in Table 1 define eligibility of sources included in this scoping review. Publications were included if all, or a clear subset of, participants were selected for the study on the basis of a T2D diagnosis with an allowance for comorbidities. Abstracts that indicated use of research-grade activity monitors to objectively measure the physical activity of participants with T2D were included. Studies that exclusively reported steps or step counts measured by accelerometers or pedometers were excluded. Conference abstracts and other sources that did not have the full text available were also excluded. In accordance with the average age of diabetes diagnosis [17], a mean age threshold of ≥50 years was implemented. Full-text articles were included if authors used specific accelerometer thresholds to categorize physical activity by intensity.

**Table 1:** Screening eligibility criteria.

Screening stage	Inclusion criteria
Abstracts	Participants selected for study on basis of type 2 diabetes diagnosis Research-grade (nonpedometer) accelerometers used to objectively measure physical activity Source is not conference abstract and full text is available Physical activity not tracked exclusively as steps or step counts Study sample has mean age ≥50 years
Full-text articles	Metabolic equivalents of task or counts-per-minute cut points reported/cited by authors and used to categorize physical activity intensity

### Search Strategy

First, a limited pilot search of PubMed and Web of Science was conducted using the Medical Subject Headings terms “accelerometry” and “diabetes mellitus, type 2,” and all related entry terms from the database. Two reviewers individually selected and analyzed 10 abstracts at random to identify recurrent keywords. Subsequently, a comprehensive search was conducted across five sources including PubMed, Web of Science, Embase, Engineering Village, and the ActiGraph Corp website research database. Finally, the reference lists of all full-text articles eligible for the review were scanned for additional original sources of evidence. Full details regarding the search strategy for each database can be found in Multimedia Appendix 1.

The PubMed search contained the major terms “accelerometry,” “diabetes mellitus, type 2,” and “exercise” as well as all related subentry terms. Results were controlled using the filter “Middle Aged + Aged: 45+” to maximize efficiency during the screening process given the age inclusion criterion stated in Table 1. All records published prior to June 23, 2020, were included, as this was the most recent date the database was accessed. The Embase search was conducted using the terms “accelerometer,” “diabetes mellitus type 2,” and “physical activity” as well as all of the related terms that automatically populate when selected. The filters “middle aged,” “aged,” and “very elderly” were used to maximize efficiency for the screening process given the age criterion in Table 1. All records published prior to July 6, 2020, were included in the screening and selection process. The Web of Science search was conducted using the complete list of terms searched in PubMed and included all articles published prior to July 12, 2020, when this search was concluded. In Engineering Village, a platform featuring multiple engineering databases such as Compendex and Inspec, records were searched using “diabetes,” “accelerometer or accelerometry or actigraph or actigraphy,” and “exercise or physical activity,” and were limited to journal articles published prior to July 12, 2020. ActiGraph, LLC produces several models of wearable activity and sleep monitors, which have been used in numerous clinical trials. The ActiGraph Corp website has a research database with various publications mentioning the use of these activity and sleep monitors. After completing the comprehensive search of the major databases mentioned hitherto, we conducted a final search for records in the ActiGraph Corp website’s research database page under the category “diabetes.” We screened all of the abstracts filed under this category with a publication date prior to July 12, 2020. Across all databases, only articles published in English were considered for inclusion.

### Source of Evidence Screening and Selection

Two reviewers independently screened all abstracts and full texts for inclusion using the predefined criteria, seeking consensus or the opinion of a third reviewer in cases of disagreement. Initially, the title, name of first author, publication year, and database name of all identified records were collected using Excel (Microsoft Corporation). Abstracts were then screened against our criteria. In cases where abstracts met the first four inclusion criteria from Table 1 but did not clearly state the age of participants, reviewers consulted the full text to confirm full eligibility. Abstract information was organized in Excel to track their original source and avoid redundant screening of duplicate records. Afterward, full texts of available abstracts were accessed and assessed for eligibility. Finally, the reference lists of all eligible full-text articles were scanned to identify any additional studies that could be included in our final pool of eligible articles. The complete screening spreadsheet is in Multimedia Appendix 2.

### Data Extraction

Two reviewers independently performed data extraction from full-text articles using a form created in Excel, with one reviewer extracting data and the other verifying it (Multimedia Appendix 3). We collected the following variables: author or authors, publication year, country, study aims and design, participant demographics, accelerometer type, placement, epoch length, total wear time, cut points, and physical activity outcomes/results. Information relevant to the accelerometer methodology that was presented in the discussion section of each study was also collected. Reviewers accessed cited articles or supplementary material if prompted by authors and necessary for data extraction.

### Synthesis and Presentation of Results

Extracted data were tabulated based on frequency distributions for the following variables: study design, accelerometer type, device placement, epoch length, total wear time, and cut points used. Study aims were consolidated into broad themes and participant demographic data were summarized and presented as a narrative. A qualitative description follows all tabulated results to relate the findings to the objectives of this review.

## Results

### Search Results

**Figure 1:**
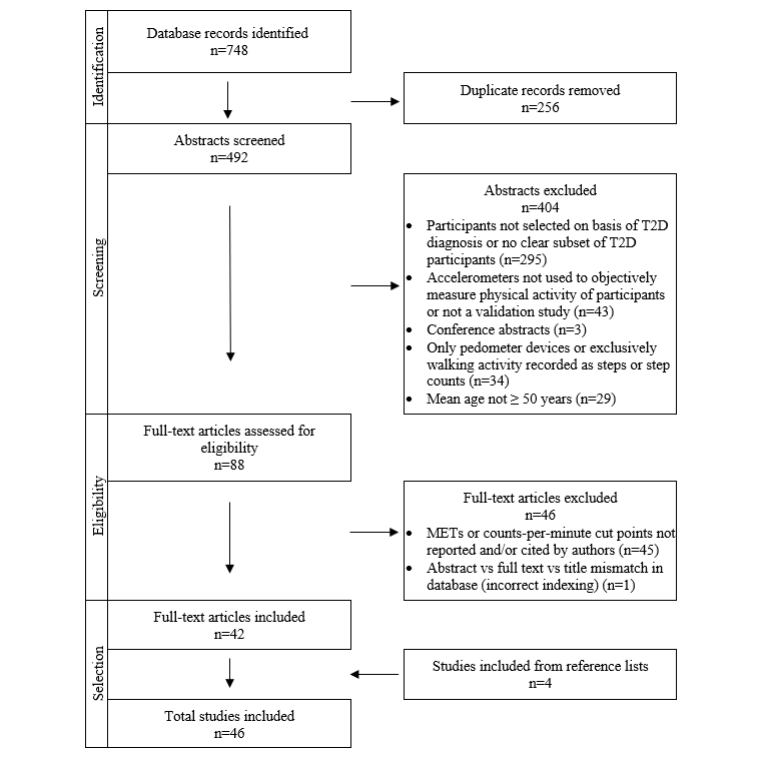
PRISMA-ScR (Preferred Reporting Items for Systematic Reviews and Meta-Analyses Extension for Scoping Reviews) flowchart of screening and selection process with reasons for elimination. MET: metabolic equivalent of task; T2D: type 2 diabetes.

The scoping review search yielded 748 records across the five databases (158 in PubMed, 201 in Embase, 309 in Web of Science, 36 in Engineering Village, and 44 in the ActiGraph Corp website). After removal of duplicates and subsequent screening of the remaining 492 abstracts, 88 full-text articles were assessed for eligibility. Of those, 42 articles met our inclusion criteria. The reference lists of these articles were then searched for additional sources of evidence, yielding 4 new articles. Ultimately, 46 articles were retained and analyzed (Figure 1 [18]).

### Source of Evidence Characteristics

Complete data extraction details are available in Multimedia Appendix 4. Most sources were cross-sectional studies (n=24), followed by RCTs (n=13), calibration/validation studies (n=2), and prospective longitudinal cohort studies (n=2). The remaining studies were correlational, mixed methods, descriptive, pretest to posttest, and case-control (Table 2). Article publication years ranged from 2008 to 2020. Of the research published in the United States, all but 2 studies had an even distribution of male and female participants, exceptions included Vanden Bosch et al [19], which enrolled only middle-aged female participants, and Whipple et al [20] whose participants were mostly male participants (80%). Among the 11 of 13 American studies that reported race/ethnicity data, 1 was comprised of at least 50% African American or Black participants [21], while the remaining 10 featured a predominantly White study population. Our age criterion captured 26 study samples with a mean age of 50 to 59.9 years, 18 samples with a mean age of 60 to 69.9 years, and 2 samples with a mean age of 70 to 79.9 years.

**Table 2:** Distribution of sources by study design (N=46).

Study design	Study, n (%)
Cross-sectional	24 (52)
Randomized controlled trial	13 (28)
Calibration/validation	2 (4)
Prospective longitudinal cohort	2 (4)
Mixed methods	1 (2)
Descriptive	1 (2)
Case control	1 (2)
Pre-to-post	1 (2)
Correlational	1 (2)

### Accelerometer Parameters

Selected RCTs examined the effect of various interventions on either physical activity (n=11) or on diabetes control (n=2), while non-RCTs similarly evaluated associations of physical activity (or lack thereof) on physical health; 2 studies conducted accelerometer calibration/validation. From an assortment of 16 different accelerometers identified across the literature, the ActiGraph was the most popular brand with its triaxial GT3X (n=10) and uniaxial GT1M (n=10) models being equally favored in 22% of studies (Table 3).

Researcher-dependent parameters for accelerometer data collection and analysis are presented in Table 4. In most studies, the accelerometer device was secured on the participant’s waist (n=23, 50%) during active data collection. Alternative placement options included the hip, wrist, chest, and on a neck strap. A total of 29 (63%) studies collected movement data in 60-second epochs (data acquisition intervals), and 23 (50%) required participants to wear the accelerometer for 7 days during data collection. Corresponding to the observed preference for ActiGraphs, various versions of the ActiLife software were used to conduct data reduction and analysis.

**Table 3:** Accelerometer devices used across research studies (N=46).

Accelerometer	Number of axes	Study, n (%)	Studies
GT3X, ActiGraph, LLC; Pensacola, FL	Triaxial	10 (22)	Poppe et al [22], Júdice et al [23], Mathe et al [24], Welch et al [25], Garcia et al [26], Castonguay and Miquelon [27], Britto et al [28], Do et al [29], Hamer et al [30], de Moura et al [31]
GT1M, ActiGraph, LLC; Fort Walton Beach, FL	Uniaxial	10 (22)	Vanden Bosch et al [19], Winkler et al [32], Eakin et al [33], Eakin et al [34], Cooper et al [35], Falconer et al [36], Healy et al [37], Goode et al [38], Lee et al [39], Falconer et al [40]
AM7164, ActiGraph, LLC; Pensacola, FL	Uniaxial	6 (13)	De Greef et al [41], Evenson et al [42], Lopes et al [43], De Greef et al [44], Loprinzi and Pariser [45], Loprinzi and Ramulu [46]
MyWellnessKey, Technogym; Cesena, Italy	Uniaxial	4 (9)	Balducci et al [47], Balducci et al [48], Balducci et al [49], McGinley et al [50]
ActiGraph, Manufacturing Technology, Inc; Fort Walton Beach, FL	Uniaxial	2 (4)	Allen et al [51], Allen et al [52]
HJA-350IT, Omron Healthcare; Kyoto, Japan	Triaxial	2 (4)	Miyamoto et al [53], Miyamoto et al [54]
RT3 Accelerometer, StayHealthy; Monrovia, CA	Triaxial	2 (4)	Unick et al [55], Jakicic et al [56]
Actiheart, CamNtech; Cambridge, United Kingdom	Triaxial	2 (4)	Guo et al [57], Cichosz et al [58]
Polar AW200 Activity Watch, Polar Electro Oy; Kempele, Finland	Uniaxial	1 (2)	Karjalainen et al [59]
MT-KT01, Terumo; Tokyo, Japan	Triaxial	1 (2)	Miyauchi et al [60]
GT9X Link, ActiGraph; Pensacola, FL	Triaxial	1 (2)	Wooldridge et al [61]
Actiwatch-Score, Philips Respironics; Bend, OR	Multidimensional	1 (2)	Fritschi et al [21]
GT3X+, ActiGraph, LLC; Fort Walton Beach, FL	Triaxial	1 (2)	Sardinha et al [62]
Fitbit Charge HR, Fitbit Inc; San Francisco, CA	Triaxial	1 (2)	An et al [63]
GT3X-BT, ActiGraph, LLC; Pensacola, FL	Triaxial	1 (2)	Whipple et al [20]
Active Style Pro HJA-750C, Omron Healthcare; Kyoto, Japan	Triaxial	1 (2)	Nishida et al [64]

**Table 4:** Parameters for accelerometer data collection and analysis (N=46).

Protocol variables		Studies, n (%)
**Device placement**		
	Waist	23 (50)
	Hip	14 (30)
	Wrist	4 (9)
	Unknown	3 (7)
	Neck strap	1 (2)
	Chest	1 (2)
**Epoch length (seconds)**		
	15	2 (4)
	30	2 (4)
	60	29 (63)
	Not reported or not applicable to device	13 (28)
**Total wear time (days)**		
	<1	1 (2)
	3	3 (7)
	4	2 (4)
	5	3 (7)
	6	3 (7)
	7	23 (50)
	10	2 (4)
	14	2 (4)
	16	1 (2)
	21	1 (2)
	28	1 (2)
	84	1 (2)
	127	1 (2)
	180	1 (2)
	183	1 (2)
**Analysis software**		
	ActiLife (various versions)	11 (24)
	SAS (various versions)	9 (20)
	SPSS (various versions)	9 (20)
	STATA (various versions)	4 (9)
	KineSoft (Saskatoon, SK)	3 (7)
	MAHUffe Analyser (various versions)	3 (7)
	ActiGraph (DOS RIU256K.EXE)	2 (4)
	BI-LINK, Omron Healthcare, Kyoto, Japan	1 (2)
	JMP Ver. 11.0.0 (SAS Institute, Japan)	1 (2)
	MATLAB (MathWorks)	1 (2)
	MeterPlusTM (Santech, San Diego, CA)	1 (2)
	MyWellnessKey online portal	1 (2)
	SAS Programs for Analyzing NHANES 2003-2004 Accelerometer Data (National Cancer Institute)	1 (2)
	Respironics Actiware (Philips Respironics, Bend, OR)	1 (2)
	StayHealthy	1 (2)
	Not reported	1 (2)

### Cut Points and Data Interpretation

This scoping review included studies in which cut points for METs or counts per minute were applied to accelerometer-measured physical activity with the aim of categorizing movement as sedentary, light physical activity (LPA), moderate physical activity (MPA), vigorous physical activity (VPA), very vigorous physical activity (VVPA), moderate-to-vigorous physical activity (MVPA), and nonlocomotive physical activity. Table 5 contains all cited cut points; some authors referenced more than one set of cut points for their data analysis. A total of 9 articles used one or more thresholds derived from the 2011 Compendium of Physical Activity, a comprehensive codebook standardizing self-reported energy expenditure into MET intensity levels [65]. These absolute cut points quantify physical activity into sedentary behavior (1.0-1.5 METs), LPA (1.6-2.9 METs), MPA (3.0-5.9 METs), VPA (6.0-8.9 METs), and VVPA (≥9.0 METs) [66,67].

The Freedson et al [67] cut points relate to the aforementioned MET intensity ranges by the regression equation shown in Multimedia Appendix 5. Per Freedson et al [67], the aforementioned MET values for LPA, MPA, and VPA correspond to <1952 counts per minute, 1952-5724 counts per minute, and ≥5725 counts per minute, respectively. Almost half (n=20) of the studies included in this scoping review used the Freedson et al [67] cut points. Of those using an ActiGraph device, 18 also used the study by Freedson et al [67] to categorize activity intensity. The Matthews et al [68] cut point for sedentary time (<100 counts per minute) compliments Freedson et al [67] but differentiates sedentary behavior from LPA when used concurrently.

The Troiano et al [69] cut points, cited in 8 articles, categorize LPA as 100-2019 counts per minute, MPA as 2020-5998 counts per minute, and VPA as ≥5999 counts per minute. Of the remaining cut points identified through this scoping review, the ones calibrated by Lopes et al [43] are intended to reflect the expected metabolic capabilities of older adults with T2D with overweight or obesity. The thresholds for sedentary-light, light-moderate, and moderate-vigorous activity are 200, 1240, and 2400 counts per minute, respectively (see Multimedia Appendix 5 for the corresponding regression equation). The Lopes et al [43] cut points were applied in 1 additional study to determine the effects of an aerobic exercise intervention on physical activity levels in adults with T2D [31].

**Table 5:** Frequency distribution for cited cut points (N=46).

Source		Cut points	Studies, n (%)
**Counts per minute**			
	Freedson et al [67]	SED^a^ and LPA^b^: <1952	20 (43)
		MPA^c^: 1952-5724	
		VPA^d^: ≥5725	
	Matthews et al [68]	N/A^e^	11 (24)
	Troiano et al [69]	LPA: 100-2019	8 (17)
		MPA: 2020‐5998	
		VPA: ≥5999	
	Matthew [8]	LPA: 100-759	2 (4)
		MVPA^f^: ≥760	
	Lopes et al [43]	SED: ≤200	2 (4)
		LPA: 201-1239	
		MPA:1240-2399	
		VPA: ≥2400	
	Unknown source	SED: <200	1 (2)
		LPA: 202-1800	
		MVPA: >1800	
	Unknown source	MVPA: ≥2296	1 (2)
	Aguilar-Farías et al [70]	SED: ≤200	1 (2)
		LPA: 201-2690	
		MPA: 2691-6166	
		VPA: 6167-9642	
		VVPA^g^: ≥9643	
	Spierer et al [71], Crouter et al [72], Harvey et al [73]	SED: <20	1 (2)
		MVPA: >20	
	Hamer et al [30]	SED: ≤199	1 (2)
		LPA: 200-1998	
		MVPA: >1999	
**Metabolic equivalent of task**			
	Ainsworth et al (if used) [65]	SED: <1.5	9 (20)
		LPA: 1.5-2.9	
		MPA: 3-5.9	
		VPA: ≥6	
	Unknown source	MPA: 2-5	1 (2)
		VPA: >5	
	Oshima et al [74]	N-LPA^h^: ≥1.5	2 (4)

^a^SED: sedentary.

^b^LPA: light physical activity.

^c^MPA: moderate physical activity.

^d^VPA: vigorous physical activity.

^e^N/A: not applicable.

^f^MVPA: moderate-to-vigorous physical activity.

^g^VVPA: very vigorous physical activity.

^h^N-LPA: nonlocomotive physical activity.

## Discussion

### Principal Findings

Among 46 peer-reviewed publications that met our inclusion criteria, this scoping review revealed that an assortment of accelerometer cut points have been used by researchers to categorize physical activity intensity for middle-aged and older adults with T2D. We found that the ActiGraph models GT3x and GT1M were most frequently used for data collection, and the Freedson et al [67] cut points were most applied for analysis. Of the 2 validation/calibration studies identified, one [43] calibrated new ActiGraph cut points with our population of interest.

### Challenges in Accelerometer Use

The current literature identifies certain limitations to accelerometer-based cut points that may lead to an underrepresentation of meaningful changes in physical activity. For example, the Freedson et al [67] thresholds were originally validated with a sample of healthy young adults (mean age 24.8, SD 4.2 years for male participants and 22.9, SD 3.8 years for female participants). In comparison, the mean age of study participants in this review ranged from 50 to 79.9 years. The Freedson et al [67] cut points are derived from the simultaneous measurement of activity counts with an accelerometer and metabolic cost with open circuit spirometry during graded treadmill exercises. Therefore, when these cut points are used to assess physical activity interventions among individuals with lower physical capacity, such as middle-aged or older adults with T2D, they potentially underestimate time spent performing MVPA. Miller et al [75] found that, when expressed relative to an individual’s maximal aerobic capacity, MPA intensity levels are not consistent across all ages of adulthood. Results revealed a substantial difference between the amount of MPA captured for older (60-69 years) and younger (20-29 years) age groups, 2847-5376 counts per minute versus 4573-6786 counts per minute, respectively. Thus, LPA intensity for a 20- or 40-year-old can be considered MPA intensity for a 60-year-old. With almost half of the studies in our scoping review favoring the application of the Freedson et al [67] cut points for data analysis, it is necessary to consider the implications of using these cut points to interpret physical activity data in populations with ages and abilities different than those of the original population used to validate them.

Multiple authors in our review [21,24,27,42] also noted that data collection is limited by device location, and accelerometers are therefore unable to capture the entire spectrum of human movement, potentially causing a misrepresentation of physical activity quantity and intensity (eg, a waist-worn device may record upper body strength training as sedentary behavior). In addition, the lack of standardization in data collection protocols, as evidenced in our review by the variety of choices for device placement, epoch length, and total wear time, makes it difficult to compare physical activity data across studies. Furthermore, as previously exemplified with the Freedson et al [67] thresholds, the choice of cut points directs data interpretation. Some authors [24,25,55,59,76] recognize that absolute cut points are not sensitive to variations in individual fitness level, which is affected by age and health status. Physical activity data for adults who are older and have chronic diseases like T2D may be disproportionately affected by the use of absolute cut points originally validated with data from their younger and healthier counterparts.

### Limitations

Our scoping review is subject to limitations. First, relevant articles may have been omitted due to our inclusion criteria and our specific research questions. For example, populations with lower physical fitness and chronic disease other than T2D were excluded. In addition, our studies were limited to those published in English and available at the time of screening. We eliminated abstracts exclusively reporting step counts; however, walking studies were acceptable if cut points were used to determine intensity. Second, we recognize an inherent potential for bias because we consulted the ActiGraph Corp website research database as the final step of our search strategy. A total of 25 unique articles were identified through this website but were subsequently excluded from analysis after failing to meet our screening criteria. Therefore, our final analysis was not impacted by the decision to search the website. Third, statistical analysis extends beyond the framework of scoping reviews, so we did not perform a quality assessment of the best cut points to use or methodology to follow for future physical activity interventions in our population of interest.

### Conclusion

The use of appropriate accelerometer cut points for the measurement of physical activity in middle-aged and older adults with T2D is critical in guiding clinically meaningful public health recommendations for T2D management in these populations. While the Lopes et al [43] cut points have been documented and were used in 1 other study, more interventional research applying them is warranted to confirm if they reflect clinically significant changes in physical activity for adults with diabetes and comorbidities other than overweight/obesity. Alternatively, relative cut points, rather than absolute cut points, could provide a more appropriate determination of physical activity based on individual fitness. Ultimately, there remains a need to develop and further test diabetes-specific cut points that can precisely and accurately assess physical activity interventions and guide public health recommendations.

## Appendix

Multimedia Appendix 1 Narrative description of scoping review search strategy.

Multimedia Appendix 2 Scoping review screening results.

Multimedia Appendix 3 Data extraction form.

Multimedia Appendix 4 Scoping review data extraction.

Multimedia Appendix 5 Regression equations.

Multimedia Appendix 6 Completed PRISMA-ScR (Preferred Reporting Items for Systematic Reviews and Meta-Analyses Extension for Scoping Review) checklist.

## Abbreviations

LPA: light physical activity
MET: metabolic equivalent of task
MPA: moderate physical activity
MVPA: moderate-to-vigorous physical activity
RCT: randomized controlled trial
T2D: type 2 diabetes
VPA: vigorous physical activity
VVPA: very vigorous physical activity
