# Supplementary material for: The Physical Activity Assessment of Adults With Type 2 Diabetes Using Accelerometer-Based Cut Points: Scoping Review
Source: Interact J Med Res. 2022 Sep 6;11(2):e34433. doi: 10.2196/34433 (PMC9490541; doi:10.2196/34433)
Supplement: Multimedia Appendix 1 [file ijmr_v11i2e34433_app1.docx]

**Pilot Search:**

A limited pilot search in **PubMed** and **Web of Science** was conducted using the Medical Subject Headings (MeSH) terms ‘accelerometry’ and ‘diabetes mellitus, type 2’ and all related entry terms from the MeSH database:

(("Accelerometry"[Mesh] OR accelerometer OR accelerometers OR actigraph OR actigraphs OR actigraphy) AND ("Diabetes Mellitus, Type 2"[Mesh] OR Diabetes Mellitus, Noninsulin-Dependent OR Diabetes Mellitus, Ketosis-Resistant OR Diabetes Mellitus, Ketosis Resistant OR Ketosis-Resistant Diabetes Mellitus OR Diabetes Mellitus, Non Insulin Dependent OR Diabetes Mellitus, Non-Insulin-Dependent OR Non-Insulin-Dependent Diabetes Mellitus OR Diabetes Mellitus, Stable OR Stable Diabetes Mellitus OR Diabetes Mellitus, Type II OR NIDDM OR Diabetes Mellitus, Noninsulin Dependent OR Diabetes Mellitus, Maturity-Onset OR Diabetes Mellitus, Maturity Onset OR Maturity-Onset Diabetes Mellitus OR Maturity Onset Diabetes Mellitus OR MODY OR Diabetes Mellitus, Slow-Onset OR Diabetes Mellitus, Slow Onset OR Slow-Onset Diabetes Mellitus OR Type 2 Diabetes Mellitus OR Noninsulin-Dependent Diabetes Mellitus OR Noninsulin Dependent Diabetes Mellitus OR Maturity-Onset Diabetes OR Diabetes, Maturity-Onset OR Maturity Onset Diabetes OR Type 2 Diabetes OR Diabetes, Type 2 OR Diabetes Mellitus, Adult-Onset OR Adult-Onset Diabetes Mellitus OR Diabetes Mellitus, Adult Onset))

Two reviewers individually selected and analyzed ten abstracts at random to identify recurrent keywords that should be added to the search terms. ‘Physical Activity’ and ‘Exercise’ were identified as additional keywords to include while searching. The pilot search was completed on June 10, 2020.

**Comprehensive Search:**

1. The **PubMed** search was based on the following search terms:

(("Accelerometry"[Mesh] OR accelerometer OR accelerometers OR actigraph OR actigraphs OR actigraphy) AND ("Diabetes Mellitus, Type 2"[Mesh] OR Diabetes Mellitus, Noninsulin-Dependent OR Diabetes Mellitus, Ketosis-Resistant OR Diabetes Mellitus, Ketosis Resistant OR Ketosis-Resistant Diabetes Mellitus OR Diabetes Mellitus, Non Insulin Dependent OR Diabetes Mellitus, Non-Insulin-Dependent OR Non-Insulin-Dependent Diabetes Mellitus OR Diabetes Mellitus, Stable OR Stable Diabetes Mellitus OR Diabetes Mellitus, Type II OR NIDDM OR Diabetes Mellitus, Noninsulin Dependent OR Diabetes Mellitus, Maturity-Onset OR Diabetes Mellitus, Maturity Onset OR Maturity-Onset Diabetes Mellitus OR Maturity Onset Diabetes Mellitus OR MODY OR Diabetes Mellitus, Slow-Onset OR Diabetes Mellitus, Slow Onset OR Slow-Onset Diabetes Mellitus OR Type 2 Diabetes Mellitus OR Noninsulin-Dependent Diabetes Mellitus OR Noninsulin Dependent Diabetes Mellitus OR Maturity-Onset Diabetes OR Diabetes, Maturity-Onset OR Maturity Onset Diabetes OR Type 2 Diabetes OR Diabetes, Type 2 OR Diabetes Mellitus, Adult-Onset OR Adult-Onset Diabetes Mellitus OR Diabetes Mellitus, Adult Onset)) AND ("Exercise"[Mesh] OR Exercises OR Physical Activity OR Activities, Physical OR Activity, Physical OR Physical Activities OR Exercise, Physical OR Exercises, Physical OR Physical Exercise OR Physical Exercises OR Acute Exercise OR Acute Exercises OR Exercise, Acute OR Exercises, Acute OR Exercise, Isometric OR Exercises, Isometric OR Isometric Exercises OR Isometric Exercise OR Exercise, Aerobic OR Aerobic Exercise OR Aerobic Exercises OR Exercises, Aerobic OR Exercise Training OR Exercise Trainings OR Training, Exercise OR Trainings, Exercise) Filters: Middle Aged + Aged: 45+ years

As seen above, the filter “Middle Aged + Aged: 45+ years’ was applied to the results in order to improve efficiency and relevance of the records screened. All records published prior to June 23, 2020 were included in the screening and selection process. This was the most recent date the database was accessed.

2. The **Embase** search was based on three main search terms and their nested/related terms that automatically populate when selected (see screenshot below). The filters ‘middle aged’, ‘aged’, and ‘very elderly’ were applied to maximize efficiency for the screening process. All records published prior to July 6, 2020 were included in the screening and selection process.

**
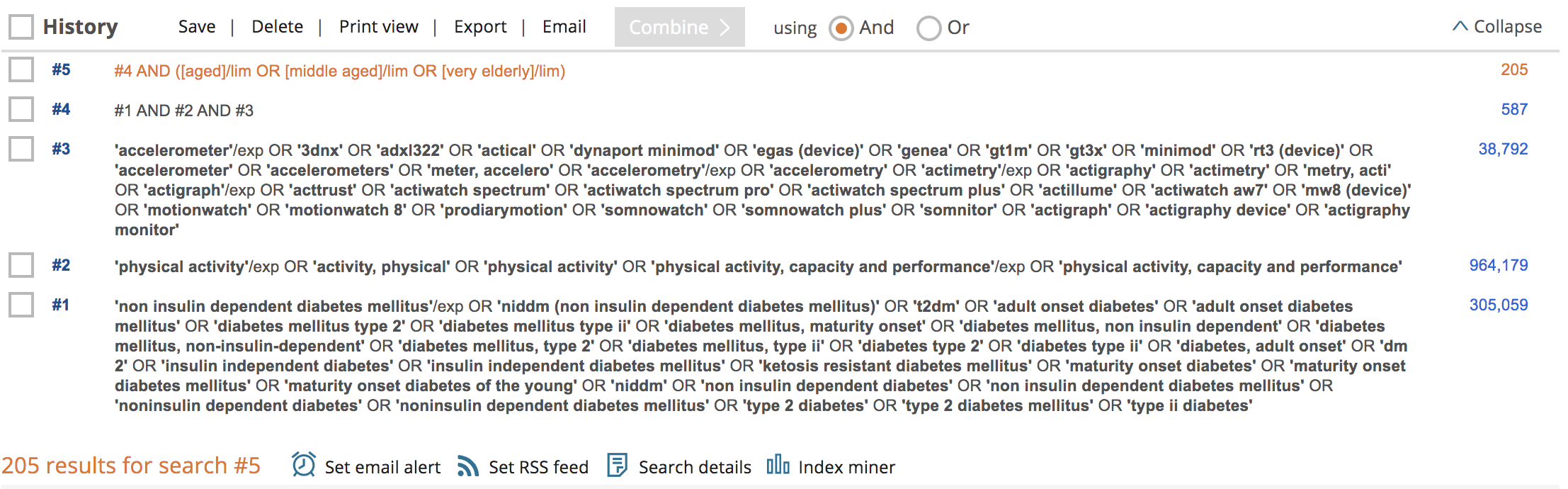
**

3. The **Web of Science** search was conducted using the complete list of terms searched in PubMed and included all articles published prior to July 12, 2020 when the database was accessed for the last time. No filters were applied on the results, contrasting the search in PubMed.

4. The **Engineering Village** search, which was limited to journal articles using the filter provided, was conducted using the following search terms:

(diabetes) AND (accelerometer OR accelerometry OR actigraph OR actigraphy) AND (exercise or physical activity)

All records published prior to July 12, 2020 were included in the screening process, as this was the last time the database was accessed.

5. The **ActiGraphcorp.com** website was accessed last for thoroughness, as it contains a research database in which publications are filed under major topics such as ‘diabetes’. All records categorized/tagged under ‘diabetes’ and published prior to July 12, 2020 were screened for eligibility.

**Reference List Search:**

The reference lists of all full text articles initially eligible for the review were scanned for additional sources of evidence. At the conclusion of this search, 4 additional articles were found to be eligible for the review.

During the entire search and screen process, only records published in English were considered for inclusion.
