# Supplementary material for: The Physical Activity Assessment of Adults With Type 2 Diabetes Using Accelerometer-Based Cut Points: Scoping Review
Source: Interact J Med Res. 2022 Sep 6;11(2):e34433. doi: 10.2196/34433 (PMC9490541; doi:10.2196/34433)
Supplement: Multimedia Appendix 5 [file ijmr_v11i2e34433_app5.docx]

**Appendix IV.** Regression Equations for Energy Expenditure

| Regression Equations | |
| --- | --- |
| Freedson et al, 1998 | MET = 1.439008 + (0.000795 * counts·min^-1^); r^2^ = 0.82; SEE = ± 1.12 METs |
| Lopes et al, 2009 | MET = 1.388400490262 + 0.001312683420044 (counts·min^–1^); r = 0.867 |
